# Association of Peri-Implant Keratinized Mucosa Width and Mucosal Thickness with Early Bone Loss: A Cross-Sectional Study

**DOI:** 10.3390/jcm13071936

**Published:** 2024-03-27

**Authors:** Zeynep Tastan Eroglu, Dilek Ozkan Sen, Elif Oncu

**Affiliations:** 1 Department of Periodontology, Faculty of Dentistry, Necmettin Erbakan University, Beyşehir Caddesi, Bağlarbaşı Sk., 42090 Konya, Turkey; dilekozkan19@hotmail.com; 2 Panoroma Ankara Private Oral and Dental Health Clinic, 06510 Ankara, Turkey; oncu.elif@hotmail.com

**Keywords:** dental implants, crevicular fluid, keratinized mucosa width, mucosal thickness, alveolar bone loss, microRNAs

## Abstract

**Objective:** The objective of this study was to evaluate the effects of keratinized mucosa width (KMW) and mucosal thickness (MT) around dental implants on marginal bone loss (MBL). The evaluation was performed one year after loading by comparing clinical, radiographic, and biochemical parameters. **Methods:** The study included 87 implants in 87 patients undergoing regular follow-ups without hard or soft tissue augmentation one year after loading. Clinical measurements included plaque index (PI), gingival index (GI), bleeding on probing (BoP), probing depth (PD), KMW, and MT. MBL was assessed with periapical radiography. The peri-implant crevicular fluid (PICF) was analyzed for tumor necrosis factor-alpha (TNF-α), receptor activator of nuclear factor-kB ligand (RANKL), osteoprotegerin (OPG), and microRNA-27a. **Results:** The MBL of implants with thin MT (<2 mm) was higher than that of implants with thick MT (≥2 mm) (*p* < 0.05). A significant negative correlation (r: −0.217) was established between MT and MBL. No significant association was found between KMW and MBL (*p* > 0.05). No significant associations was found between KMW and MT with TNF-α, RANKL, OPG and RANKL/OPG (*p* > 0.05), with the exception of increased microRNA-27a levels in implants with KMW ≥ 2 mm (*p* < 0.05). **Conclusions:** Implants with a thick MT had a lower MBL. There may be an association between adequate KMW and high miRNA-27a levels. The relationship between MBL and miRNA-27a remains unclear.

## 1. Introduction

Dental implants are a reliable option for treating missing teeth [1]. For dental implants to remain successful over the long term, it is vital that marginal bone levels are preserved [2]. Surgical trauma [3], implant design [4], the type and location of the implant–abutment connection interface [5,6], mucosa thickness (MT) [7,8], keratinized mucosa width (KMW), [9] buccal wall thickness [10], occlusal overload [11], and peri-implantitis [12] have all been identified as potential contributors to marginal bone loss (MBL) surrounding dental implants.

The peri-implant phenotype refers to the specific morphological and dimensional features that determine the clinical appearance of the tissues that surround and support osseointegrated implants. The peri-implant phenotype consists of two components: soft tissue and hard tissue [13]. The hard tissue component of the peri-implant phenotype is the peri-implant bone thickness, while the peri-implant soft tissue component is constituted by the peri-implant MT, the KMW, and the supracrestal tissue height. The term is not limited to the buccal and labial sites but includes the lingual and palate peri-implant regions. Similar to the periodontal phenotype, the peri-implant phenotype is distinct in each location and can undergo alterations over time due to various environmental conditions [13].

MT, one of the soft tissue components of the peri-implant phenotype, is vital to peri-implant health and aesthetic outcomes [14]. However, most research investigating the correlation between initial bone modeling and soft tissue thickness has concentrated on supracrestal tissue height. Very little research has examined the impact of horizontal mucosal thickness on marginal bone loss. One study has shown that implants with a decreased MT are linked to increased clinical attachment loss [15]. However, soft tissue grafting methods can increase MT, leading to a reduction in peri-implant MBL [7].

In many studies, the presence of keratinized tissue surrounding the implant has been reported to have a positive effect on meeting aesthetic expectations and protecting peri-implant health [16]. Despite the conflicting opinions expressed in the literature, studies have indicated that a wide keratinized tissue band plays a crucial role in providing a protective barrier against mechanical damage during oral hygiene procedures, particularly in patients with significant bone and soft tissue loss. Additionally, it helps to preserve the junctional epithelium during functional movements of the mucosa, thereby preventing mucogingival stress and contributing to the maintenance of peri-implant tissue health [14,17,18,19,20,21].

Peri-implant crevicular fluid (PICF), an oral fluid, has been suggested as a viable supplementary diagnostic tool, similar to gingival crevicular fluid (GCF), due to its non-invasive nature and ease of collection. Several studies have assessed PICF using clinical data to investigate the relationship between peri-implant soft tissue and MBL. These studies showed that implants with inadequate KMW have higher levels of prostaglandin E2 [14] and tumor necrosis factor-alpha (TNF-α) [18] and a higher ratio of the receptor activator of nuclear factor-kB ligand (RANKL) to osteoprotegerin (OPG) [22].

MicroRNAs (miRNAs), which have been recognized as crucial regulators of bone hemostasis, affect osteoclastogenesis by directly regulating osteoclast activity, signaling intermediates, or through negative feedback loops. In contrast, they control osteogenesis through positive feedback loops [23]. Therefore, they may mediate alveolar bone resorption in peri-implantitis and periodontitis [24]. Although the role of miRNAs in periodontal and peri-implant diseases is still uncertain, their potential as diagnostic tools is promising due to their detectability in oral fluids [25,26].

In a histological study on dogs, miRNA-27a correlated negatively with TNF-α and induced osteogenesis [27]. However, upon reviewing the literature, it was evident that no studies had investigated the association of this miRNA with peri-implant soft tissue.

Based on this knowledge, this cross-sectional study aimed to evaluate the effects of MT and KMW on MBL through clinical, radiological, and immunological parameters. The null hypothesis was that there is no significant relationship between MT, KMW, and MBL, as assessed through clinical, radiological, and immunological parameters.

## 2. Materials and Methods

### 2.1. Study Design and Sample Selection

This study was conducted by recalling individuals whose single missing tooth had been treated with an implant-supported fixed restorations in the Periodontology Department of Necmettin Erbakan University, Faculty of Dentistry. Our study was approved by the Non-Pharmacological and Non-Medical Device Research Ethics Committee (2021/04-48) of Necmettin Erbakan University, Faculty of Dentistry, and it was conducted according to the 1975 Declaration of Helsinki, as revised in 2013. The study protocol was recorded on ClinicalTrials.gov (NCT05640284). The study fully complied with the Strengthening the Reporting of Observational Studies in Epidemiology (STROBE) checklist.

**Figure 1:**
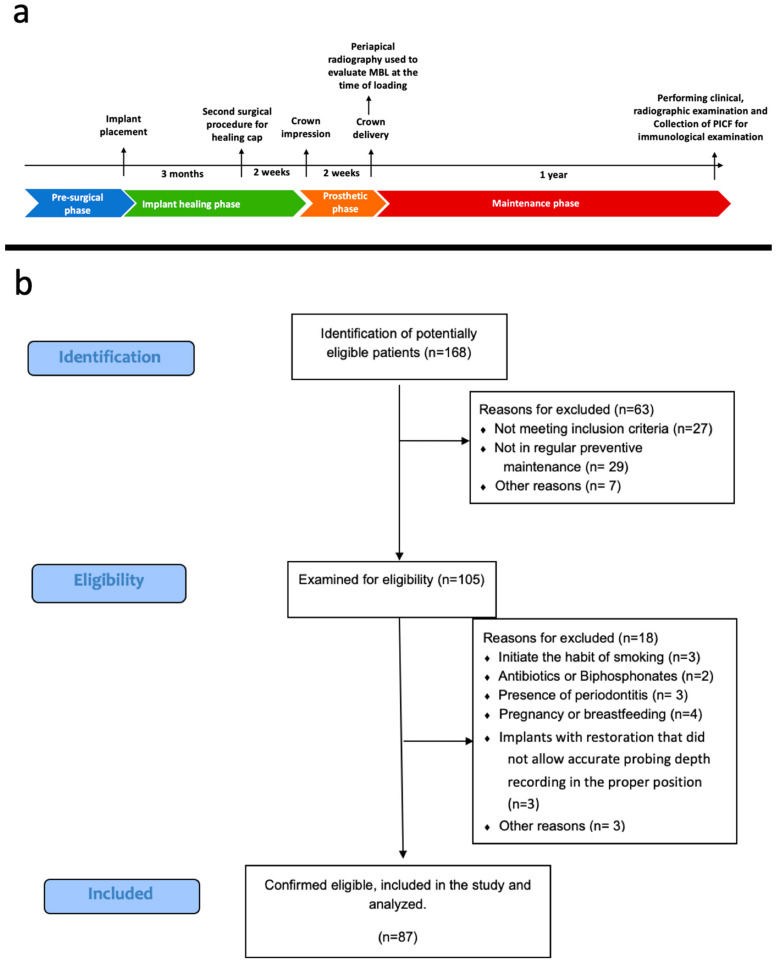
(**a**) Study timeline. (**b**) Flowchart of patient allocation and intervention.

Patient recruitment started in November 2022 and was completed by the end of January 2023. Written and verbal consent was acquired from all participants who agreed to participate in the study. The clinical procedures and evaluations were conducted within the same time frame. Implants included in the study were bone-level implants with sand blasted with large grits with an acid etched surface, submerged placement, ti-base abutment, and screw abutment connection, all put in place by the same periodontologist (E.O.) between September and November 2021. A summary of this study timeline is depicted in Figure 1a.

The inclusion and exclusion criteria are summarized in Table 1. The inclusion and exclusion criteria were applied during the implant placement, implant loading, and this assessment. Figure 1b shows the experimental flowchart for this study.

Each implant examined in the study was inserted in adherence to the manufacturer’s recommended protocol. Second-stage surgery was performed after a healing period of 3 months. Restorative procedures were initiated after 2 weeks of connecting the healing abutment. After the delivery of the prostheses, all of the patients were instructed regarding implant hygiene. The patients were then included in a maintenance program at the study center with a 3-month recall interval.

Personal information (age, gender) and implant localization (maxilla, mandible) information were recorded for each patient.

### 2.2. Peri-Implant Clinical Parameters

A calibrated periodontist (Z.T.E.) used a 0.5 mm-diameter plastic periodontal probe (PCPNU-15, Hue-Friedy, Chicago, IL, USA). The gingival index (GI) [28] was used to assess the peri-implant soft tissue status, and the plaque index (PI) [29] was used to assess plaque levels. The peri-implant pocket depth (PD) was measured at six points around the implant (mesio-facial, mid-facial, disto-facial, mesio-lingual, mid-lingual, and disto-lingual), and bleeding on probing (BoP) was recorded. BoP was assessed by determining the existence of visual indications of inflammation and bleeding during a gentle 15-s probing. Suppuration was evaluated by observing visual signs of suppuration following the probing.

#### KMW and MT Measurements

Peri-implant KMW and MT were measured for each implant. KMW was calculated at the midfacial as the distance in millimeters from the free mucosal margin to the mucogingival junction and classified as adequate (≥2 mm) or inadequate (<2 mm) [13].

Buccal mucosal thickness measurements were conducted after the administration of topical and/or local anesthetic (2% lidocaine with 1:100,000 epinephrine), which was introduced into the vestibular depth to prevent any unintentional increase in gingival thickness at the designated measurement sites. Buccal mucosal thickness was measured at a distance of 1 mm from the soft tissue margin at the midfacial location that corresponded to the same location for probe visibility assessment by the same clinical investigator. An endodontic file (30 K-file; Dentsply, Tulsa, OK, USA) was inserted transgingivally until contact with the tooth structure was felt. Flowable composite (3M ESPE, St. Paul, MN, USA) was applied around the file, on the external surface of the gingiva, and cured (SmartLite, DENTSPLY, Charlotte, NC, USA)to create a fixed reference point [30], and the distance between the file tip and the fixed reference point was measured using a digital caliper (Alpha Tools Digital Caliper, Mannheim, Germany), with a resolution of 0.05 MT being classified as thin (<2 mm) or as thick (≥2 mm) [13].

### 2.3. Radiographic Examination and Measurements

**Figure 2:**
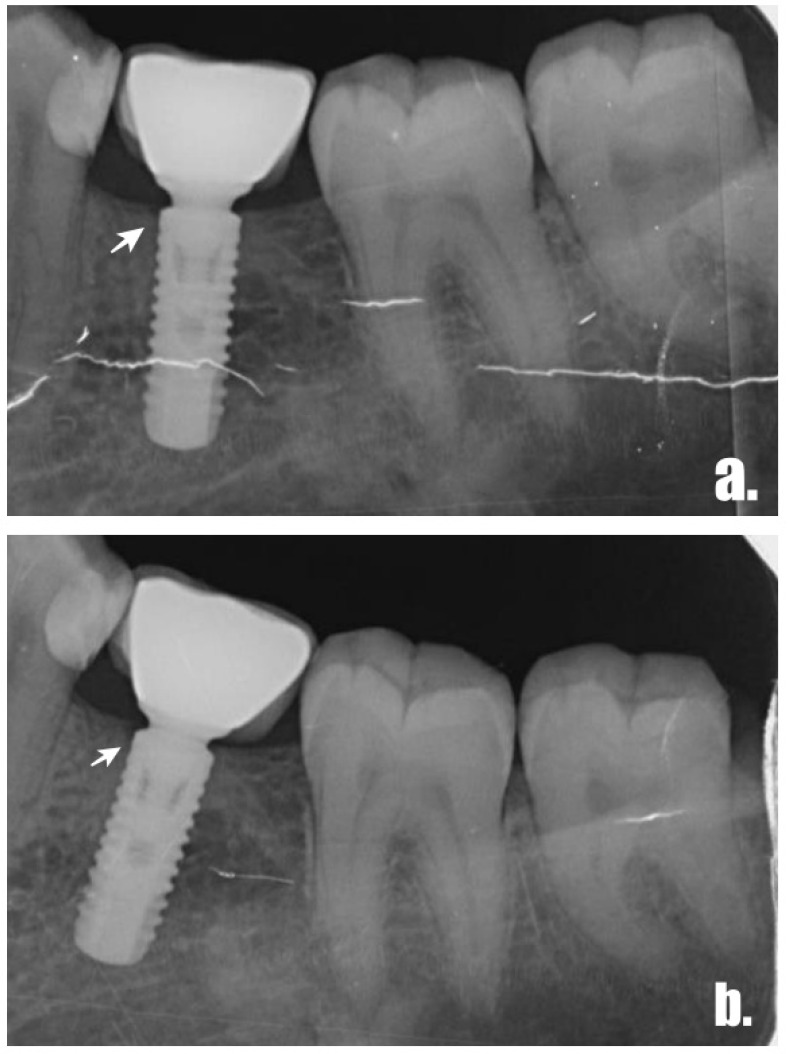
(**a**) Crestal bone level after prosthetic restoration and (**b**) after 1-year follow-up. Arrows: the most marginal bone-to implant contact level.

One examiner (D.Ö.S), who was blind to the clinical features of the implants, performed the radiographic evaluation of the periapical radiographs (Figure 2). Intraoral periapical radiographs were acquired utilizing a plastic film holder and the paralleling technique following the clinical evaluation. Computer software (Turcasoft Dent, Samsun, Turkey) was used to evaluate the radiographs’ digital pictures. Periapical radiographs were obtained during treatment planning, following implant surgery (initial), at restoration delivery, and one year after the prosthetic loading. To determine MBL, a single independent calibrated inspector (D.Ö.S) carried out linear measurements on each periapical radiograph from the most mesial and distal point of the implant–abutment junction to the crest bone using the program (Turcasoft, Samsun, Turkey). The magnification of the radiographs was corrected according to the clinical data (height and width) for each implant. A simple mathematical calculation was performed to calibrate and recalculate each linear MBL measurement according to the radiographic image size. For the MBL evaluation, the measurements were compared with those in radiographs taken after prosthesis delivery.

One experienced and calibrated examiner (Z.T.E.) conducted all clinical measurements (GI, PD, BOP, KMW, MT), while another experienced and calibrated examiner (D.Ö.S) performed all radiographic measurements. To ensure accurate and consistently reproducible measurements, a calibration procedure was undertaken until a 90% agreement coefficient was achieved. Intra-examiner reproducibility was evaluated in three patients, demonstrating that the variability between repeated measurements (baseline and at 24 h) was less than 3% [31].

### 2.4. Sample Collection and Determination

#### 2.4.1. Collection of Peri-Implant Crevicular Fluid (PICF)

**Figure 3:**
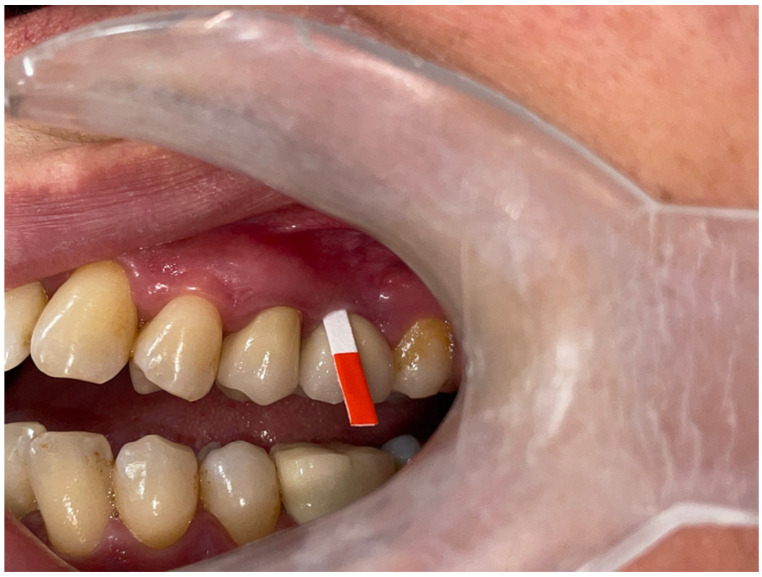
Collection of peri-implant crevicular fluid.

PICF samples were obtained one year after the implant was loaded. The supragingival plaque was eliminated, the sampling area was isolated using sterile cotton rolls, and the site was carefully dried with air to prevent any contamination during the collection procedure. Standardized paper strips (PerioPaper strips, Oraflow Inc., Smithtown, NY, USA) were inserted into the pocket until they formed a resistance. The paper strips remained in the peri-implant pocket for 30 s (Figure 3). The procedures were repeated in cases where the strips were contaminated with saliva or blood. PICF volumes were determined as described previously [18,32]. Strips were placed into coded micro centrifuge tubes and stored at −80 °C until the analysis day.

#### 2.4.2. Determination of TNF-α, RANKL, and OPG

Before biochemical analyses, 0.5% bovine serum albumin was placed in Eppendorf tubes containing paper strips and 200 μL of PBS solution. PICF was separated from the strips via centrifugation at 5000× *g* at 4 °C for 6 min. Methylcellulose strips were cut, and their contents were assimilated by adding 800 µL-sterilized PBS. The samples were examined using commercially procurable ELISA kits (Human TNF-α ELISA kit Elabscience, Houston, TX, USA; Human RANKL ELISA kit Elabscience, Houston, TX, USA; and Human OPG ELISA kit Elabscience, Houston, TX, USA), following the manufacturer’s instructions. The minimum level of detection or the lower level of detection (LLD) values for the ELISA kits are as follows: 7.81 pg/mL for TNF-α, 15.63 pg/mL for RANKL, and 0.16 ng/mL for OPG.

#### 2.4.3. Determination of miRNA-27a

First, miRNAs were isolated for miRNA-27a analysis. In the isolation procedure, samples were incubated with a miRNA isolation kit (Bio Basic, Markham, ON, Canada) for 5–10 min at room temperature. They were vortexed for 30 s by adding 0.2 mL of chloroform and centrifuged at 12,000× *g* at 4 °C for 10 min. The supernatant formed following centrifugation (approximately 540 μL) was transferred to a clean 1.5 mL RNase-free centrifuge tube and centrifuged according to the manufacturer’s instructions. The RNA solution eluted at −80 °C was recorded. The miRNA obtained was converted to cDNA using a cDNA isolation kit (miRNA All-In-One cDNA Synthesis Kit, Applied Biological Materials Inc., Richmond, BC, Canada). The final volume was studied as 20 µL by the kit’s study protocol of the kit. Master mix (BrightGreen miRNA qPCR MasterMix Kits, Applied Biological Materials Inc., Canada), primary mixes (miRNA-27a RT-PCR kit, Applied Biological Materials Inc., Canada), and U6-2 housekeeping gene (U6-2 Primers, Applied Biological Materials Inc., Canada) were prepared following the manufacturer’s recommendations to ensure the amplification of cDNAs regarding the reference gene and to mark the related regions. The prepared reference gene, real-time PCR mixes, and target gene real-time PCR mixes were subjected to the RT-PCR procedure in line with the manufacturer’s protocol using an RT-PCR system (Light Cycler 96 system, Rotkreuz, Switzerland) with appropriate cDNAs.

### 2.5. Statistical Analysis

The implants were categorized based on mucosal thickness, and marginal bone loss was utilized as the variable. Through a power analysis conducted using data from a previous study, it was determined that, with a power of 80% and a Type 1 error rate of 5%, there should be 17 participants in each group [15]. Statistical software (SPSS version 21, IBM, Armonk, NY, USA) was used within the scope of the study. Mean standard deviation values were used for the continuous variables examined, and percentage values were used for the categorical data. Within the framework of the research, the data for 87 participants were evaluated. In this study, the normality assumption was checked with the Shapiro–Wilk test. According to the results obtained, the distribution was determined to be normal, and it was found appropriate to perform parametric tests and examinations. In the data analysis, the chi-squared test was used to compare categorical data. To compare categorical groups with continuous variables, an independent samples *t*-test was used. Pearson’s correlation analysis was employed to investigate the correlation between binary and continuous variables. The statistical significance value was accepted as *p* < 0.05 in this study.

## 3. Results

### 3.1. Study Sample

The study evaluated a total of 87 participants, including 57 females and 30 males, who had a total of 87 implants. The mean age of the participants was 48.1 ± 7.7 years.

### 3.2. Peri-Implant Clinical Parameters

The mean values for the peri-implant clinical parameters were: PD 2.36 ± 0.77 mm, PI 1.01 ± 0.61, and GI 1.26 ± 0.75. The mean MT was 1.90 ± 0.89 mm, and the mean KMW was 2.67 ± 1.68 mm. Out of the 87 implants that were examined, 68 of them had an adequate KMW (≥2 mm) display, while 19 implants had an inadequate KMW (<2 mm). Out of the total number of implants analyzed, 55 exhibited a thick MT (≥2 mm), while 32 implants displayed a thin MT (<2 mm). Table 2 presents the association between the KMW and MT of implants across various variables. No significant association was observed between participant age, gender, implant location, PI, GI, and PD values, and the KMW and MT values for the implants (*p* > 0.05). Implants with adequate KMW (≥2 mm) were observed to have a statistically significantly thicker MT (thick MT: 70.6%; thin MT: 29.4%) than those with inadequate KMW (<2 mm) (thick MT: 36.8%; thin MT: 63.2%) (*p* = 0.008). Implants with thick MT (≥2 mm) (adequate KMW: 87.3%; inadequate KMW: 12.7%) were observed to have statistically significantly, more adequate KMW (≥2 mm) than those with thin MT (<2 mm) (adequate KMW:62.5%; inadequate KMW: 37.5%) (*p* = 0.008).

### 3.3. Radiographic Examination

Mean MBL values were statistically higher for implants with thin MT (<2 mm) than for implants with thick MT (≥2 mm) (*p* < 0.05) (Table 2). In the correlation analysis, a significant negative correlation between MT and MBL was revealed; MT increased as MBL decreased (*p* < 0.005) (Table 3). No significant association was observed between KMW and the MBL of the implants (*p* > 0.05).

### 3.4. Immunological Parameters

A statistically significant relationship was not observed between the KMW and MT status of the investigated implants and the levels of TNF-α, RANKL, OPG, and RANKL/OPG (*p* > 0.05) (Table 4).

When the association between KMW and miRNA-27a was examined, it was found that the quantity of miRNA-27a was statistically significantly higher in implants with adequate KMW (*p* < 0.05).

Although not statistically significant, higher miRNA-27a values were observed in implants with thicker MT (*p* > 0.05) (Table 4).

## 4. Discussion

In this research conducted with individuals enrolled in the post-implantation supportive maintenance program, the association of MT and KMW with MBL and with immunological parameters was evaluated one year after the loading of the implants. The radiographic examination revealed that implants with thick MT had less MBL. There was a negative correlation between MT and MBL, but no significant correlation between KMW and MBL. To our knowledge, this is the first study to evaluate the relationships between KMW, MT, and miRNAs. Implants with adequate KMW exhibited a statistically significant increase in miRNA-27a levels.

The eligibility criteria for the study were determined following a comprehensive research review to optimize the potential for quantitative data analysis. The KMW and MT were both designated a threshold of 2 mm [13]. However, although 2 mm is the most frequently used threshold value for research, this value is nevertheless arbitrarily determined [33].

In this study, the total amount of PICF was assessed. Studies stated that the total amount of GCF is a better indicator than its concentration, and it has been argued that the concentration is directly affected by the sample volume. The total amount yields a more objective result [34].

The majority of studies evaluating the impact of soft tissue thickness on initial bone remodeling have focused on supracrestal tissue height, measured before implant placement. In a study classifying patients into thin (<3 mm) and thick (≥3 mm) groups, as based on supracrestal tissue height, marginal bone loss was assessed at 1, 2, 3, 6, and 12 months after implant placement. Significantly less bone loss was observed in the thick group compared to the thin group [35]. Another study assessed marginal bone loss in the second and twelfth months and found greater bone loss in implants with short supracrestal tissue height at both these time points [36]. Because this study was conducted retrospectively, the evaluation of supracrestal soft tissue thickness was not possible as it had not been recorded.

However, more consensus has yet to be reached on the minimum MT required to achieve predictable long-term functional and aesthetic outcomes and to minimize MBL [37]. In one of the limited studies evaluating the impact of horizontal MT on marginal bone loss, after one year of loading, no significant difference in MBL was observed between implants with <1 mm and ≥1 mm MT. However, it was noted that implants with thin MT exhibited greater clinical attachment loss compared to those with thicker mucosa [15]. Most studies in this field have focused on the effect of increasing MT for aesthetic purposes. A recent systematic review has elucidated that soft tissue grafting performance results in significantly less interproximal MBL over time following MT acquisition [7]. In this study, when implants with thin and thick MT were compared, implants with thick MT exhibited less MBL. A negative correlation was observed between MT and MBL.

At present, the literature offers differing results regarding the impact of KMW on MBL. Numerous studies indicate that implants with less than 2 mm KMW have a higher MBL [21,38]. Conversely, many other studies have revealed no difference in MBL in relation to “adequate” and “inadequate” KMW [17,39,40]. In our study, no significant differences in MBL were identified between implants with adequate or inadequate KMW.

Information on peri-implant bone thickness and short-term bone loss after implant placement is limited. In one study on this subject, buccal bone thicknesses were evaluated using cone-beam computed tomography subsequent to implant placement. Implants demonstrating 2 mm or more of buccal bone thickness were found to be associated with less marginal bone loss [41]. In another study, it was reported that regions with at least 2 mm of peri-implant bone thickness and approximately 0.5 mm apical to the implant crest at the time of implant placement exhibited lower rates of vertical bone loss between 6 to 8 months post-implant placement and slightly lower implant failure rates [42]. All implants evaluated in the present study had a minimum bone thickness of 2 mm after the implant placement procedure.

While various diagnostic methods are currently employed to identify inflammation in the peri-implant region, it is acknowledged that detecting inflammatory conditions before clinical symptoms manifest is essential for conservative treatment approaches [43]. PICF, which is osmotically derived from the gingival vascular plexus, is regarded as analogous to gingival crevicular fluid. It is believed that PICF can be used to identify early inflammatory changes around implants due to its content of inflammatory mediators [17,43].

Several studies have assessed PICF with clinical data to investigate the relationship between peri-implant soft tissue and MBL. Zigdon and Machtei assessed PICF prostaglandin E2 levels in dental implants with adequate or inadequate KMW [14]. Boynuegri et al. conducted a longitudinal study to examine the impact of KMW on peri-implant clinical and immunological parameters over 12 months [18]. PI, GI values, and levels of TNF-α were found to be significantly higher with implants that had inadequate KMW. Another examination revealed an elevated RANKL/OPG ratio in the PICF of implants that had inadequate KMW six months after the insertion of the implant [22]. In this study, No significant associations was found between KMW and MT with TNF-α, RANKL, OPG and RANKL/OPG.

In recent years, there has been increased interest in studying microRNAs in periodontal and peri-implant disease. In most investigations, miRNAs have been examined by invasive means in gingival tissue [27,44,45]. Therefore, the miRNA results obtained in this study have not been adequately discussed in previous studies.

A study investigating the relationship between miRNA-27a and peri-implantitis in dogs showed that an increase in miRNA-27a might induce osteogenesis by reducing TNF-α. It was reported that a high TNF-α concentration induced the downregulation of miRNA-27a and suppressed osteogenic differentiation around the implant during middle and late stages of peri-implantitis [27]. These findings suggest that miR-27a may play an important role in the maintenance and stability of dental implants. Solakoğlu et al. observed an increase in serum miRNA-27a levels during the bone healing phase in patients undergoing bone grafting compared to their pre-operation levels [46]. They suggested that these findings indicate the high diagnostic potential of miRNAs’ plasma levels for detecting clinically relevant bone resorption activities before they lead to alveolar bone loss. In a study conducted on mice, miRNA-27a was shown to decrease osteoclastic differentiation [47]. In this study, miRNA-27a was observed to be statistically significantly higher in implants with adequate KMW. Nevertheless, no association was observed between miRNA-27 and MT, TNF-α, RANKL, OPG, and RANKL/OPG.

Prior research has demonstrated a significant association between miRNA-27a and subcutaneous adipose tissue, visceral fat adipose tissue, and body mass index (BMI) [48]. Additionally, it is known that miRNA-27b is involved in polycystic ovary syndrome (PCOS) [49]. Nevertheless, this study did not collect data on the participants’ BMI or on their diagnoses of PCOS, which is a limitation of this research.

Another limitation of the present study was the relatively small number of implants included in the analysis since the inclusion criteria were to keep the numbers limited (*n* = 87).

The supracrestal tissue height of the evaluated implants was not measured during the implant surgery. Therefore, this parameter could not be assessed in the study. This situation is a limitation of retrospective design of the study. However, prospective studies on this subject are needed, and supracrestal tissue height should also be considered in future studies. Furthermore, initial KMW and MT measurements were not taken, and PICF samples were not collected when the implants were loaded.

Accepting its limitations, this study is the first to evaluate the association between KMW, MT, and miRNA-27a.

## 5. Conclusions

In conclusion, less MBL was observed in implants with thick MT, and as MT increased, a significant decrease in MBL was noted. Furthermore, there appears to be a potential correlation between adequate KMW and high levels of miRNA-27a. However, no significant association was found between miRNA-27 and MT, TNF-α, RANKL, OPG, and RANKL/OPG. The relationship between MBL and miRNA-27a remains uncertain. Further research is required with a larger sample size, using a prospective longitudinal study design, measuring MT non-invasively, and evaluating PICF and MBL.

## Figures and Tables

**Table 1:** Inclusion and exclusion criteria.

Inclusion Criteria
(1) Age: 18–65 (2) Single implant because of tooth missing in the maxillary or mandibular premolar or first molar region (3) Implants with adjacent distal and mesial teeth (4) Residual bone height of ≥9 mm and bone width of ≥6 mm before implant placement (5) Implants loaded one year previously (6) Non-smokers or quit smoking at least six months before implant placement (7) Maintaining routine controls
Exclusion Criteria
(1) Use of soft or hard tissue grafts during any period before or after the placement and loading of the implants (2) Implants with a buccal bone thickness of less than 2 mm after implant placement (3) Pregnancy or breastfeeding (4) Uncontrolled medical conditions (e.g., diabetes mellitus with hemoglobin A1c ≥ 7.0) (5) Conditions known to change bone metabolism (e.g., diabetes, osteopenia, osteoporosis, and hyperparathyroidism) (6) Use of oral or intravenous bisphosphonates (7) Use of systemic antibiotics and/or anti-inflammatory drugs within the last three months (8) Alcohol or drug addiction (9) Having a history of periodontitis and having undergone any periodontal treatment in the last 6 months (10) Implants with restoration that did not allow accurate probing depth recording in the proper position

**Table 2:** Comparison of KMW and MT statuses according to different variables.

Variables	KMW Inadequate (<2 mm) (*n* = 19)	KMW Adequate (≥2 mm) (*n* = 68)	*p*	MT Thin (<2 mm) (*n* = 32)	MT Thick (≥2 mm) (*n* = 55)	*p*
**Age ^†^**	49.32 ± 9.96	47.88 ± 7.02	0.477	49.44 ± 9.30	47.47 ± 6.62	0.255
**Gender ^‡^**						
**Female**	11 (58.0%)	47 (69.1%)	0.246	23 (71.8%)	12 (60.0%)	0.152
**Male**	8 (42.0%)	21 (30.8%)		9 (28.1%)	8 (40.0%)	
**Location ^†^**						
**Maxilla**	4 (21.1%)	29 (42.6%)	0.066	10 (31.3%)	23 (41.8%)	0.100
**Mandibula**	15 (78.9%)	39 (57.4%)		22 (68.7%)	32 (58.2%)	
**Mean MBL (mm) ^†^**	0.46 ± 0.73	0.59 ± 0.70	0.456	0.57 ± 68	0.13 ± 0.32	0.012 *
**PI ^†^**	1.00 ± 0.67	1.02 ± 0.56	0.923	1.06 ± 0.67	0.98 ± 0.53	0.539
**GI ^†^**	1.02 ± 0.56	1.11 ± 0.66	0.301	1.38 ± 0.71	1.20 ± 0.78	0.299
**PD (mm) ^†^**	2.10 ± 0.66	2.43 ± 0.79	0.098	2.40 ± 0.88	2.33 ± 0.71	0.687

Abbreviations: KMW, keratinized mucosa width; MT, mucosal thickness; MBL, marginal bone loss; PI, plaque index; GI, gingival index; PD, pocket depth. * *p* < 0.05, ^†^ test: independent samples *t*-test, ^‡^ test: chi-squared test.

**Table 3:** Correlation between keratinized tissue width, mucosal thickening, bone loss, and biochemical markers.

	MT	KMW	TNF-α	RANKL	OPG	RANKL/OPG	miRNA-27a	Mean MBL
MT	1	0.522 *	−0.097	0.020	0.077	−0.020	−0.047	−0.217 *
KMW		1	0.023	−0.036	−0.007	0.009	−0.186	−0.170
TNF-α			1	0.567 *	0.565 *	−0.117	−0.033	0.065
RANKL				1	0.654 *	0.241 *	0.001	0.083
OPG					1	−0.339 *	−0.009	−0.068
RANKL/OPG						1	−0.123	0.211 *
MiRNA-27a							1	−0.155
Mean MBL								1

Abbreviations: KMW, keratinized mucosa width; MT, mucosal thickness; MBL, marginal bone loss; TNF-α, tumor necrosis factor-alpha; RANKL, receptor activator of nuclear factor-kB ligand; OPG, osteoprotegerin; miRNA, micro ribonucleic acid, * r: 0.522, r: 0.567, r: 0.565, r: 0.654, r: 0241, r: 0.339, r: 0.217, r: 0.211 *p* < 0.05, Pearson’s correlation test.

**Table 4:** The correlation between KMW and MT status and TNF-alpha, RANKL, OPG, RANKL/OPG, and miRNA-27a.

	**KMW Adequate (≥2 mm) (*n* = 68)**	**KMW Inadequate (<2 mm) (*n* = 19)**	***p* ^†^**
**TNF-α (pg/mL)**	8.24 ± 5.59	6.33 ± 2.89	0.155
**RANKL (pg/mL)**	10.75 ± 7.39	8.91 ± 5.80	0.321
**OPG (pg/mL)**	0.11 ± 0.09	0.10 ± 0.08	0.47
**RANKL/OPG**	106.70 ± 56.97	110.42 ± 45.90	0.794
**miRNA-27a**	183.71 ± 764.65	1.09 ± 1.36	0.001 *
	**MT Thick (≥2 mm) (*n* = 55)**	**MT Thin (<2 mm) (*n* = 32)**	***p* ^†^**
**TNF-α (pg/mL)**	7.38 ± 3.70	8.60 ± 7.02	0.290
**RANKL (pg/mL)**	10.39 ± 6.93	10.26 ± 7.45	0.935
**OPG (pg/mL)**	0.11 ± 0.09	0.11 ± 0.08	0.837
**RANKL/OPG**	109.94 ± 60.35	103.34 ± 43.28	0.589
**miRNA-27a**	219.97 ± 834.82	1.81 ± 3.60	0.354

Abbreviations: KMW, keratinized mucosa width; MT, mucosal thickness; MBL, marginal bone loss; TNF-α, tumor necrosis factor-alpha; RANKL, receptor activator of nuclear factor-kB ligand; OPG, osteoprotegerin; miRNA, micro ribonucleic acid, * *p* < 0.05, ^†^ test: independent samples.

## Data Availability

The data that support the findings of this study are available from the corresponding author (Z.T.E.) upon reasonable request.
